# Editorial: Alterations in the Sound Localization Pathway Related to Impaired Cocktail-Party Performance

**DOI:** 10.3389/fnins.2022.902197

**Published:** 2022-04-25

**Authors:** Elizabeth A. McCullagh, Leonard K. Kaczmarek, Daniel J. Tollin, Achim Klug

**Affiliations:** ^1^Department of Integrative Biology, Oklahoma State University, Stillwater, OK, United States; ^2^Department of Pharmacology, Yale University, New Haven, CT, United States; ^3^Department of Cellular and Molecular Physiology, Yale University, New Haven, CT, United States; ^4^Department of Physiology and Biophysics, University of Colorado Anschutz Medical Campus, Aurora, CO, United States

**Keywords:** sound localization, cocktail party effect, auditory, brainstem, interaural time and intensity differences

Binaural and spatial hearing allows us to localize the source of a sound and to function in complex acoustic environments. In noisy environments we typically focus on one sound source, e.g., our conversation partner, and ignore background noises. The sound localization pathway in the auditory brainstem contributes to this ability by associating various competing sounds with their respective spatial channel. Normal hearing humans and many animals can localize and discriminate sound sources with a precision of just a few degrees. This is accomplished by comparing the interaural time difference (ITD) and interaural intensity difference (IID) that a sound creates between the two ears, which vary systematically with the location of the sound source in space. Even small alterations in this brainstem circuit can have major effects on one's ability to function in an acoustically busy environment. This special edition highlights some of these alterations in animal models and human subjects and discusses medical conditions associated with impaired hearing in noise.

## Disordered Sound Localization Performance and Mechanisms

Importantly, the alterations discussed here affect central neural circuits and are not dependent on decreased ability to detect sounds; in many cases, however, a patient might experience both peripheral and central hearing loss. A combination of alterations along the ascending auditory pathway makes diagnosis and treatment of this condition very challenging. A review by Gallun discusses the clear need for better diagnostic tools, including behavioral and neurophysiological tests to determine the specific alterations in any particular case.

Koerner et al. show the dependence and variance of physiological and behavioral measurements in a common form of age-related hearing loss alters CNS binaural circuits. Affected listeners have clinically normal hearing thresholds but struggle to understand speech in background noise. Alterations in the binaural system of these listeners cause impaired processing of temporally fast and precise binaural cues that can be detected with electrophysiological measurements that are directly related to the behavioral ability to decode binaural cues correctly. This suggests that non-invasive physiological tests can potentially be used to quantify behavioral difficulties in affected listeners.

The ability to localize sounds is also severely disrupted in autism spectrum (ASD) disorder and inherited forms of intellectual disability such as Fragile X syndrome (FXS). FXS is caused by loss of Fragile X Mental Retardation Protein, FMRP, an mRNA-binding protein that controls translation and also regulates neural excitability by binding ion channels (Wu and Kaczmarek). An overwhelming majority of patients with FXS and ASD are hypersensitive to auditory stimuli and have difficulty in distinguishing speech sounds from background noise. As reviewed by Rotschafer, these abnormalities of auditory processing can often be detected by electric and magnetic signals recorded from the cerebral cortex of humans.

One of the biological factors altered in ASD may be the speed and precision at which auditory brainstem neurons propagate action potentials. Using a mouse model of FXS, Lucas et al. demonstrate that in the medial nucleus of the trapezoid body, a key brainstem relay for transmission of both IID and ITDs, loss of FMRP reduces both the diameter of axons and the thickness of the myelin sheath. A complementary computational investigation by Li et al., modeled such changes in myelin thickness and conduction velocity in a brainstem network. They analyzed firing patterns in response to sinusoidal tones and natural sounds and calculated tuning curves for ITDs in the medial superior olive, where the timing of inputs from the two ears is compared. The combined experimental/computational studies make a strong case that, by reducing the rate at which auditory information is propagated through the brainstem, impaired myelination disrupts accurate comparisons of ITDs in FXS.

In addition to genetic mutations, experiencing incorrect binaural cues during development may impair the high level of precision required for sound localization. The finding that abnormal early sound experiences can result in binaural neurons that incorrectly code for spatial location even in adulthood (Thornton et al.), underscores the importance of early interventions to hearing loss.

## Modulation of Brainstem Circuitry in Complex Acoustic Environments

Wu and Kaczmarek review the modulation of potassium channels in auditory brainstem neurons in response to changes in the auditory environment. They describe how insights into the role of specific channels have come from human gene mutations that impair localization of sounds in space. Additionally, they review how short-term and long-term modulation of channels maximizes the extraction of auditory information. Among these channels is the Kv3.3 potassium channel, which is further discussed by Middlebrooks and Waters who describe a family with a Kv3.3 mutation. The affected family members exhibited severe loss of sensitivity for ITDs and ILDs, which almost certainly degrades their ability to segregate competing sounds in the real-world.

Middlebrooks and Waters further review the mechanisms by which human and animal listeners segregate competing sequences of sounds from sources separated by as little as 10°. Neurons in the auditory cortex tend to synchronize selectively to one of two such competing sequences. The ability to spatially resolve these stimuli depends on the binaural and monaural acoustical cues provided in the various experimental conditions. This contrasts with a different measure of sound-source localization, the minimum audible angle, which is constant across those conditions, suggesting that the central spatial mechanisms for stream segregation differ from those for sound localization.

Finally, Auerbach and Gritton review studies of the variety of different adaptive mechanisms by which information is extracted from complex acoustic and highly variable listening conditions. These mechanisms include both “bottom-up” gain alterations in response to changes in environmental sound statistics as well as “top-down” mechanisms that allow for selective extraction of specific sound features in a complex auditory scene. The review concludes with an examination of how hearing loss interacts with these gain control mechanisms.

In summary, this special edition highlights the importance of the auditory brainstem sound localization circuit in extracting sound source locations in space using a specialized area of the brain that is fine-tuned for temporal precision. Importantly, this circuit is functionally involved in disordered spatial hearing in complex conditions such as FXS, ASD, and aging among others.

## Author Contributions

All authors listed have made a substantial, direct, and intellectual contribution to the work and approved it for publication.

## Funding

NIH 1R15HD105231-01 (EAM); NIH DC01919, NS102239, and NS111242 (LKK); NIDCD R01-011555 and NIDCD R01-017924 (DJT and AK); and NIDCD R01-018401 (AK).

## Conflict of Interest

The authors declare that the research was conducted in the absence of any commercial or financial relationships that could be construed as a potential conflict of interest.

## Publisher's Note

All claims expressed in this article are solely those of the authors and do not necessarily represent those of their affiliated organizations, or those of the publisher, the editors and the reviewers. Any product that may be evaluated in this article, or claim that may be made by its manufacturer, is not guaranteed or endorsed by the publisher.

